# The Influence of Arthroscopic Shaver Mincing and Platelet-Rich Plasma on Chondrocytes of Intraoperatively Harvested Human Cartilage

**DOI:** 10.1177/03635465231181633

**Published:** 2023-07-14

**Authors:** Sebastian Gebhardt, Alexander Zimmerer, Peter Balcarek, Georgi I. Wassilew, Janosch Schoon

**Affiliations:** † Center for Orthopaedics, Trauma Surgery and Rehabilitation Medicine, University of Greifswald, Greifswald, Germany; ‡ ARCUS Sportklinik, Pforzheim, Germany; § Department of Trauma Surgery, Orthopaedics and Plastic Surgery, University of Göttingen, Göttingen, Germany; Investigation performed at the Center for Orthopaedics, Trauma Surgery and Rehabilitation Medicine, University of Greifswald, Greifswald, Germany

**Keywords:** chondrocytes, cartilage, mincing, AutoCart, chondrocyte isolation, arthroscopic shaver, platelet-rich plasma

## Abstract

**Background:**

Minced cartilage implantation (MCI) has seen a renaissance in recent years. In this evolved technique, human articular cartilage is harvested with an arthroscopic shaver, augmented with platelet-rich plasma (PRP), and implanted with autologous thrombin. This modified technique combines the possibility of cell-based surgical cartilage repair with a minimally invasive autologous 1-step procedure. However, evidence on cell survival and preserved function after shaver-based mincing and PRP supplementation is limited.

**Purpose:**

To evaluate the effects of arthroscopic shaver mincing and augmentation with PRP on human cartilage tissue.

**Study Design:**

Controlled laboratory study.

**Methods:**

Standardized samples were taken from 12 donors during autologous MCI. A comparison of cell outgrowth, cell viability, proliferation capacity, and ability to produce extracellular matrix–specific proteoglycans after chondrogenic redifferentiation was made between cartilage taken by curettage from the border of the cartilage defect, cartilage tissue minced by an arthroscopic shaver, and cartilage tissue minced by an arthroscopic shaver that was additionally augmented with autologous PRP.

**Results:**

There was no difference between all 3 groups in terms of cell outgrowth or proliferation capacity. Metabolic activity relative to the cell number of chondrocytes isolated from shaver-minced cartilage was higher compared with chondrocytes isolated from cartilage that was derived by curettage or shaver-minced cartilage that was augmented with PRP. After chondrogenic stimulation, the normalized proteoglycan content was higher in spheroids of cells derived from shaver-minced cartilage augmented with PRP than in spheroids of cells derived from curettage. A high correlation of cell outgrowth, proliferation capacity, and viability between isolated cells from all 3 groups taken from an individual donor was observed.

**Conclusion:**

Chondrocytes isolated from human cartilage tissue that was harvested and minced with an arthroscopic shaver remained viable and proliferative. The augmentation of shaver-minced cartilage with PRP led to the enhanced proteoglycan production of chondrogenic spheroids in vitro, pointing toward the development of a cartilage-specific extracellular matrix. This in vitro study yields promising results regarding the use of an arthroscopic shaver and augmentation with PRP in the context of MCI.

**Clinical Relevance:**

Knowledge that shaver mincing and augmentation with PRP are feasible for processing articular cartilage during MCI is highly relevant for surgical cartilage repair.

Autologous minced cartilage implantation (MCI) is an operative repair technique that is becoming increasingly popular. Mincing of autologous cartilage and reimplantation into focal cartilage defects was initially described in 1983.^1^ However, it has played a minor role in cartilage repair for decades compared with established techniques such as microfracture and autologous chondrocyte implantation (ACI).

Available research on the mincing technique has shown the advantages of sharp mincing into small fragment sizes over blunt mincing^18^ and the feasibility of using a hand-mincing device.^11^ Recently, the MCI technique was innovated by an all-autologous setup, allowing for the simplified handling and arthroscopic application of minced cartilage (AutoCart; Arthrex).^17^ The subsequent increase in its clinical use has brought MCI into focus in the scientific discourse. Thus, MCI is mentioned in recent treatment recommendations as a technique with high potential but lacking supporting scientific evidence.^13^ A recently published systematic review and meta-analysis identified 3 methodical and sufficiently designed clinical trials on MCI including 52 patients.^9^ At 12- and 24-month follow-up, multifunctional outcome scores improved compared with baseline, with no available long-term follow-up.

Several preclinical studies have documented chondrocyte proliferation, the ability for extracellular matrix production, and the production of regenerated hyaline-like cartilage from minced cartilage tissue.^5,12^ The latest approach to MCI includes the use of an arthroscopic shaver for harvesting and mincing cartilage tissue, the application of autologous platelet-rich plasma (PRP) as a potential growth-stimulating and prochondrogenic factor, and the use of autologous thrombin as a sealant.^17^ This updated technique provides advantages in handling during the operative procedure and allows for all-arthroscopic applications in most cases. However, evidence of the efficacy of this updated technique is absent. In particular, the feasibility of using an arthroscopic shaver for harvesting and mincing human cartilage tissue and the effect of adding autologous PRP are investigated insufficiently.

Thus, our in vitro study aimed to investigate the feasibility of arthroscopic shaver mincing of human cartilage tissue and the effect of the addition of PRP to shaver-minced cartilage tissue during autologous MCI.

## Methods

### Patient Recruitment and Harvest of Samples

**Figure 1.**
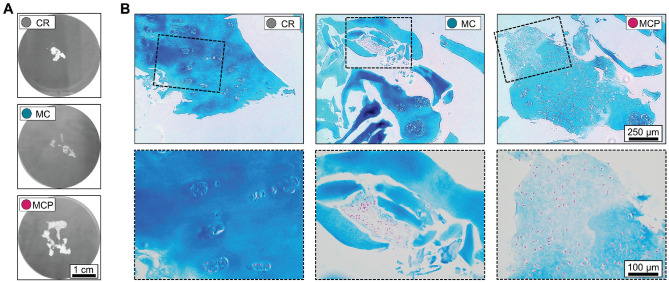
Macroscopic and microscopic comparison of differently harvested samples. (A) Representative macroscopic appearance of cartilage tissue taken by curettage (CR), after harvesting with an arthroscopic shaver (MC), and after harvesting with an arthroscopic shaver and the addition of PRP (MCP). (B) Representative Alcian blue staining of tissue sections indicates an intact cartilage matrix with embedded chondrocytes, a release of chondrocytes from the matrix with the majority of chondrocytes remaining intact, and released chondrocytes agglomerated in PRP. CR, curettage; MC, minced cartilage; MCP, minced cartilage with autologous PRP; PRP, platelet-rich plasma.

Overall, 3 standardized cartilage tissue samples were obtained from 12 consecutive patients undergoing MCI for focal cartilage defects of the knee (Table 1). Ethics approval was obtained from the local ethics committee of the University of Greifswald (BB009/21a) in accordance with the World Medical Association’s Declaration of Helsinki. All patients provided written informed consent. There were 3 different samples taken intraoperatively during MCI (Figure 1A). The first sample was taken by curettage (CR) with a sharp spoon from the border of the cartilage defect, which was necessary to create solid and sharp defect borders as generally recommended in cartilage repair surgery (CR group). The second sample was harvested from remaining cartilage tissue in the affected zone and from loose or partially damaged cartilage fragments at the border of the cartilage defect with use of an arthroscopic shaver (3-mm Sabre; Arthrex) and thus minced to pastelike cartilage tissue (MC) at the same time (MC group). The operative protocol called for a designated collection container (GraftNet; Arthrex) that was interposed in the standard suction of the shaver for the withdrawal of shaver-minced cartilage tissue. However, remains of shaver-minced cartilage would always stick to the surface of the GraftNet and could be collected directly after the operative procedure to be analyzed for this study. The third sample was shaver-minced cartilage tissue generated as described above and additionally augmented with autologous PRP (MCP group). PRP was produced simultaneously with the operative procedure. To this end, 45 mL of patient blood was drawn from the right or left antecubital region using 3 ACP Double-Syringe Systems (Arthrex). According to the manufacturer’s protocol, full blood samples were centrifuged (Horizon 24-AH; Drucker Diagnostics) to generate a supernatant of PRP and leukocyte-poor plasma in the ACP Double-Syringe Systems. The inner syringe of the double-syringe system was used to separate the plasma from the cellular portion of the sample within a closed system. The PRP gained was mixed 1:3 (vol/vol) with shaver-minced cartilage tissue and prepared for reimplantation. During reimplantation, a defect filling of 80% to 90% was aspired, as recommended for the surgical procedure described elsewhere.^20^ Processed shaver-minced and PRP-augmented cartilage tissue left in the application syringe when sufficient defect filling was achieved was used for this study.

**Table 1:** Patient and Defect Characteristics as Well as Cell Quantity Relative to Sample Weight After Primary Outgrowth^*a*^

					No. of Cells Relative to Sample Weight, n/mg		
Patient	Sex	Age, y	Defect Size, cm^2^	Defect Location	CR	MC	MCP
1	F	59	6.0	MFC	5144	5921	4722
2	F	43	1.5	MFC	2423	6866	7179
3	F	45	2.0	MFC	7815	18,056	9178
4	M	20	1.0	Retropatellar	21,467	25,113	109,130
5	F	51	2.5	MFC	549	731	1353
6	F	33	5.0	Retropatellar	428	108	431
7	M	45	4.0	Trochlea	378	247	408
8	M	28	2.0	MFC	4533	3420	4111
9	M	43	1.5	MFC	3913	1382	2464
10	M	25	2.0	MFC	3520	2873	2036
11	F	27	1.5	Retropatellar	2983	615	806
12	M	50	6.0	MFC	8549	12,987	4278

a CR, curettage; F, female; M, male; MC, minced cartilage; MCP, minced cartilage augmented with platelet-rich plasma; MFC, medial femoral condyle.

### Isolation, Cultivation, and Cryopreservation of Primary Chondrocytes

The samples, intraoperatively harvested by CR (CR group), after mincing with the arthroscopic shaver (MC group), and after mixing shaver-minced cartilage with PRP (MCP group), were weighed. After the CR samples were cut into 1-mm × 1-mm pieces with a scalpel, the samples were transferred to 6-well tissue culture plates at a weight to surface area ratio of 25 mg/10 cm^2^ and cultured in low-glucose Dulbecco’s modified Eagle medium (DMEM; PAN-Biotech) supplemented with 10% fetal bovine serum (Sigma-Aldrich), 100 U/mL penicillin (Thermo Fisher Scientific), 100 µg/mL streptomycin (Thermo Fisher Scientific), and 2 mM L-alanyl-L-glutamine (GlutaMAX; Thermo Fisher Scientific) at 37°C in a 5% CO_2_ atmosphere. Culture media were changed twice a week. Cells were detached on day 14 of primary cell culture by 0.05% trypsin containing 0.02% EDTA (PAN-Biotech), and cell numbers were quantified using the TC20 Automated Cell Counter (Bio-Rad Laboratories). Cells were seeded at a density of 1.0 × 10^3^ cells/cm^2^ and cultured until 80% confluence. The isolated chondrocytes were cryopreserved in cell culture passage 2 in low-glucose DMEM containing 12.5% human serum albumin (Biotest Pharma) and 10% dimethyl sulfoxide (AppliChem). The chondrocytes were thawed and further expanded for subsequent in vitro experiments.

### Quantification of Cell Viability and Proliferation Capacity

In cell culture passage 3, the chondrocytes were seeded on 48-well tissue culture plates at a density of 1.8 × 10^3^ cells/well. The quantification of metabolic activity as a marker for cell viability was performed by using a resazurin-based assay (PrestoBlue; Invitrogen) according to the assay manual at 24 hours after seeding (referred to as day 0), on day 4, and on day 8. Media changes were performed on day 0 and day 4. Fluorescence intensities were quantified with a multimode microplate reader (Infinite M200 PRO; Tecan) at 560 nm (excitation) and 590 nm (emission). After cell viability quantification, the individual plates were washed with phosphate-buffered saline (PBS; Bio&Sell) and frozen at −80°C until thawing to determine the proliferation capacity by DNA quantification (CyQuant Cell Proliferation Assay; Thermo Fisher Scientific) according to the assay manual. The fluorescence intensities were quantified at 485 nm (excitation) and 530 nm (emission). Population doubling was calculated by relating the relative fluorescence unit (RFU) of the CyQuant assay on day 4 and day 7 to the value on day 0 [log (RFU (d4 or d7)*/RFU* (d0))/log (2)].

### Chondrogenic Redifferentiation

Spheroids of 3 × 10^5^ cells from cell culture passage 4 were generated by centrifugation at 400×*g* for 10 minutes in deep 96-well round-bottom plates. The expansion medium was replaced by 500 µL of chondrogenic medium based on 4500-mg/L glucose DMEM supplemented with 173 µM L-ascorbic acid 2-phosphate sesquimagnesium salt, 0.1 µM dexamethasone, 0.35 mM L-proline (Sigma-Aldrich), 1 mM sodium pyruvate (PAN-Biotech), 1.25 mg human serum albumin (Biotest Pharma), 6.25 µg/mL insulin–transferrin–sodium selenite media supplement (Sigma-Aldrich), 19.1 µM linoleic acid (Sigma-Aldrich), 100 U/mL penicillin, 100 µg/mL streptomycin, and 2 mM L-alanyl-L-glutamine (GlutaMAX). Recombinant human transforming growth factor beta 1 (TGF-β1) (BioLegend) at a concentration of 10 ng/mL was added to induce chondrogenic differentiation. After centrifugation at 400×*g* for 10 minutes, the spheroids were cultured for 21 days at 37°C in a 5% CO_2_ atmosphere. Media changes were performed twice a week. On day 21, the spheroids were either washed with PBS and cryopreserved at −80°C until the quantification of proteoglycan and protein content or fixed overnight in 4% formaldehyde solution (Herbeta) and stored in PBS at 4°C until histological processing.

### Proteoglycan and Protein Quantification

A dimethylmethylene blue assay was performed to quantify the proteoglycan content of the chondrogenic spheroids. To this end, 2 spheroids of each group were pooled, milled, and chemically digested as previously described.^2,7^ Absorbance was quantified at 516 nm. The concentrations were calculated by plotting the absorbance values of the spheroid lysates against the absorbance values of standards from chondroitin sulfate sodium (Sigma-Aldrich). The protein content of the spheroid lysates was quantified by the Bradford protein assay (Pierce Coomassie Protein Assay Kit; Thermo Fisher Scientific) according to the manufacturer’s instructions.

### Histology

The cartilage samples were fixed overnight in 4% formaldehyde solution, dehydrated with an ascending series of ethanol, stored in xylene (Carl Roth) for 24 hours, and embedded in paraffin/xylene (Carl Roth) (1:1) at 65°C for 2.5 hours and subsequently in paraffin. Then, 5-µm sections were cut using a microtome (RM2255; Leica).

The chondrogenic spheroids were dehydrated with an ascending series of ethanol and xylene before embedding in paraffin and cutting into 7-µm sections. Alcian blue staining of cartilage sections and chondrogenic spheroid sections was performed after dewaxing with xylene and rehydrating with a descending series of ethanol. The rehydrated sections were placed in 3% acetic acid solution (Carl Roth) for 10 minutes, stained with 1% Alcian blue 8GX solution (Morphisto) in 3% acetic acid (Th. Geyer) for 30 minutes, counterstained with nuclear fast red (Carl Roth) for 5 minutes, and dehydrated with an ascending series of ethanol. Bright-field imaging of the stained sections was performed with a hybrid microscope (Rebel; ECHO).

### Statistical Analysis

GraphPad Prism (Version 8.4.3; GraphPad Software) was used for statistical analyses and data plotting. The investigators were not blinded to group allocation during the experiments. Sample size was not predetermined by statistical methods. Detailed information about sample sizes per group, error bars, and statistical analyses of the individual experiments are included in the figure legends. No samples or data were excluded from the analyses, and all data points are shown as individual values. The smaller sample size in the chondrogenic redifferentiation experiment is because of limiting cell yields after primary cell isolation of individual samples.

## Results

### Characterization of Samples

Overall, 3 standardized cartilage samples were taken from every donor to investigate the influence of harvesting and mincing by an arthroscopic shaver and the possible effects of augmenting shaver-minced human cartilage tissue with autologous PRP. CR samples consisted of multiple cartilage tissue fragments in the millimeter range. In comparison, MC samples had a pastelike appearance, and macroscopically, no cartilage fragments could be distinguished. However, the adhesion of pastelike cartilage tissue was low. Comparatively high adhesion and a sticky, loamy appearance were observed in MCP samples (Figure 1A).

The histological evaluation of CR samples showed intact hyaline cartilage tissue with chondrocytes in lacunae embedded in the cartilage matrix. The process of arthroscopically mincing cartilage led to partial fragmentation of the cartilage matrix and the release of embedded chondrocytes, while the majority of chondrocytes remained intact. In comparison, in the MCP group, PRP seemed to process a neomatrix-like structure embedding the previously released chondrocytes (Figure 1B).

### Comparison of Cell Outgrowth

**Figure 2.**
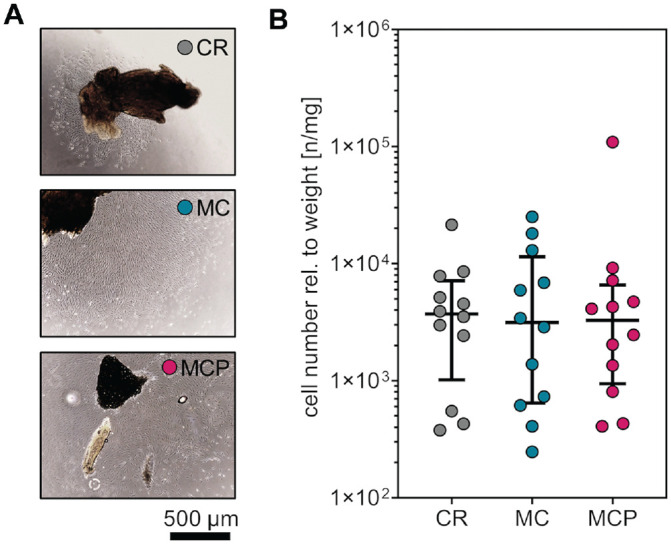
Cell outgrowth during primary cell culture. (A) Representative phase-contrast microscopy indicates cell outgrowth from cartilage samples on day 14 of primary cell culture. (B) Cell numbers normalized to the sample weight after 13 to 15 days of primary cell culture. Data are shown as median with IQR (Friedman test with Dunn post hoc test). CR, curettage; MC, minced cartilage; MCP, minced cartilage with autologous platelet-rich plasma; rel., relative.

To investigate the feasibility of using an arthroscopic shaver for harvesting cartilage tissue and to analyze the possible effects of augmenting minced human cartilage tissue with autologous PRP, cell outgrowth from shaver-minced cartilage was examined and compared with cartilage derived from CR. During primary cell passage, cell outgrowth was observed from all types of samples, irrespective of mincing or the addition of PRP (Figure 2A).

Cell numbers relative to the weight were analyzed in cell culture passage 0 after 13 to 15 days of primary cell culture. Harvesting of human cartilage tissue with an arthroscopic shaver and augmenting shaver-minced cartilage with autologous PRP did not affect in vitro cell outgrowth compared with the cell outgrowth of cartilage derived by CR (median cell numbers per weight [n/mg]: CR: 4221 [IQR, 1018-7147]; MC: 3147 [IQR, 644-11,457]; MCP: 3286 [IQR, 943-6564]) (Figure 2B).

### Comparison of Cell Viability and Proliferation Capacity

**Figure 3.**
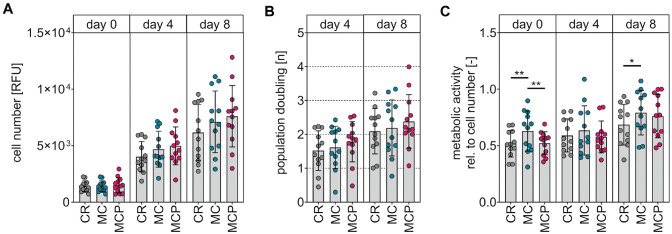
Evaluation of the proliferation capacity and cell viability of isolated chondrocytes from the differently harvested samples. (A) Cell numbers determined by DNA quantification on days 0, 4, and 8. (B) Population doubling of cells on days 4 and 8. (C) Cell viability normalized to cell numbers by metabolic activity on days 0, 4, and 8. Data are shown as mean ± SD (1-way analysis of variance with Tukey post hoc test). CR, curettage; MC, minced cartilage; MCP, minced cartilage with autologous platelet-rich plasma; RFU, relative fluorescence unit; *P < .05; **P < .01.

Metabolic activity and cell numbers were quantified to analyze whether the different harvesting methods influenced the cell viability and proliferation capacity of the isolated chondrocytes. The cell numbers of the 3 groups were compared by DNA quantification on day 0 (24 hours after seeding), day 4, and day 8. Our analyses did not indicate differences in chondrocytes isolated from differently harvested cartilage in terms of cell numbers (Figure 3A) and population doubling (Figure 3B). The metabolic activity on day 0 normalized to the corresponding cell number was found to be higher in chondrocytes isolated from cartilage from the MC group (relative mean cell viability ± SD on day 0: 0.63 ± 0.18) in comparison with chondrocytes isolated from cartilage from the MCP group (0.52 ± 0.10; *P* = .0065) and chondrocytes isolated from cartilage from the CR group (0.52 ± 0.12; *P* = .0046). In addition, the metabolic activity on day 8 normalized to the corresponding cell number was found to be higher in chondrocytes isolated from cartilage of the MC group (0.79 ± 0.20) compared with chondrocytes isolated from cartilage of the CR group (0.69 ± 0.18) (*P* = .0499). No differences were observed between any of the groups on day 4, and no difference was observed between chondrocytes from the CR group and chondrocytes from the MCP group at any time point (Figure 3C).

### Comparison of Chondrogenic Redifferentiation Potential

**Figure 4.**
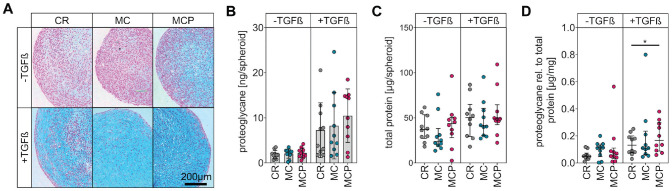
Chondrogenic redifferentiation of cells isolated from the differently harvested samples. (A) Representative images of Alcian blue–stained spheroid sections after 21 days of 3-dimensional cell culture with and without chondrogenic stimulation by transforming growth factor–beta (TGF-β). The blue color indicates glycosaminoglycans. (B) The proteoglycan quantification of spheroid lysates. Data are shown as mean ± SD (1-way analysis of variance with Tukey post hoc test). (C) The protein quantification of spheroid lysates. Data are shown as median with interquartile range (Friedman test with Dunn post hoc test). (D) Proteoglycan content normalized to protein content. Data are shown as median with interquartile range (Friedman test with Dunn post hoc test). *P < .05. CR, curettage; MC, minced cartilage; MCP, minced cartilage with autologous platelet-rich plasma; rel., relative.

Cells from all 3 groups were stimulated for chondrogenic redifferentiation in 3-dimensional cultures to analyze whether the cartilage-processing method influenced their potential to form a cartilage matrix. After TGF-β1 stimulation, representative histological sections of chondrocyte spheroids from all 3 groups accumulated glycosaminoglycans, as indicated by Alcian blue staining (Figure 4A). No differences were observed in the proteoglycan content (Figure 4B) and protein content (Figure 4C) of the spheroids generated from cells of the individual groups. Pairwise testing of donor-specific samples revealed that in 8 of 10 investigated pairs, the normalized proteoglycan content of the MCP sample was higher than that of the corresponding CR sample. Accordingly, normalization of the proteoglycan content to the protein content revealed a higher median proteoglycan proportion in chondrogenic spheroids of cells isolated from shaver-minced cartilage augmented with PRP (0.17 µg/mg [IQR, 0.09-0.30]) than in spheroids of cells isolated from cartilage derived by CR (0.13 µg/mg [IQR, 0.08-0.20]) (*P* = .0417) (Figure 4D).

### Donor-Specific Influence

**Figure 5.**
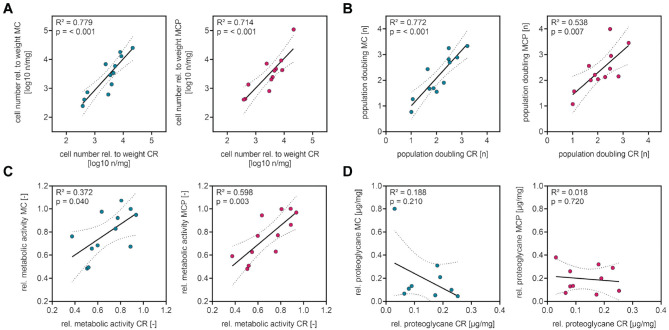
Correlation analyses indicate a donor-dependent correlation of cell outgrowth, proliferation capacity, and viability of cells from specimens obtained by the different intraoperative harvesting methods. (A) Cell outgrowth in the MC and MCP groups correlates with cell outgrowth in the CR group. Plotted is the decadic logarithm of cell numbers after primary isolation. (B) The proliferation capacity of chondrocytes in the MC and MCP groups correlates with the proliferation capacity of chondrocytes in the CR group. Plotted is the population doubling on day 8 after seeding. (C) The cell viability in the MC and MCP groups correlates with the cell viability in the CR group. Plotted is the metabolic activity on day 8 after seeding. (D) The proteoglycan content of chondrogenic spheroids in the MC and MCP groups does not correlate with the cell viability in the CR group. Plotted is the proteoglycan content of the spheroids relative to the protein content of the spheroids. Data are shown as Pearson correlation coefficient and 95% CI (dashed lines) (linear regression). *R*^2^, coefficient of determination. CR, curettage; MC, minced cartilage; MCP, minced cartilage with autologous PRP; rel., relative.

During data analyses, distinct interindividual differences in analyzed parameters between samples taken from different donors were observed. Correlation analyses of cell numbers after cell outgrowth, proliferation capacity, cell viability, and proteoglycan proportion of chondrogenic spheroids were performed to examine whether these distinct differences were caused by donor-dependent factors. The correlation analyses revealed that the number of cells outgrown from shaver-minced cartilage and from shaver-minced cartilage augmented with PRP correlated with the number of cells outgrown from cartilage that was taken by CR (Figure 5A). Moreover, the proliferation capacity (Figure 5B) and the cell viability (Figure 5C) of cells isolated from shaver-minced cartilage and from shaver-minced cartilage augmented with PRP correlated with the proliferation capacity and cell viability of cells from cartilage taken by CR. In contrast, no correlation was observed between the proteoglycan proportion of chondrogenic spheroids from shaver-minced cartilage and of chondrogenic spheroids from cartilage taken by CR or between the proteoglycan proportion of chondrogenic spheroids from shaver-minced cartilage augmented with PRP and of chondrogenic spheroids from cartilage taken by CR (Figure 5D).

## Discussion

The main findings of the present study are that samples that were harvested and minced by an arthroscopic shaver retained cell outgrowth and that cells isolated from these samples retained proliferation capacity and cell viability comparable with cartilage tissue from the same donor that was harvested by CR. Furthermore, the viability of cells derived from shaver mincing was higher compared with the viability of cells derived from CR. The addition of PRP to shaver-minced cartilage tissue did not affect cell outgrowth, cell viability, or proliferation capacity. Still, it led to a significantly higher normalized proteoglycan content compared with chondrocytes isolated from CR not augmented with PRP. This finding indicates that adding PRP enhanced the ability of chondrocytes from shaver-minced cartilage to produce a cartilage-specific extracellular matrix.

The ideal technique for mincing cartilage tissue has been studied extensively: Sharp devices were superior to blunt devices in fragmenting cartilage tissue before transplantation, as blunt devices led to zones of cell necrosis.^16^ Furthermore, the degree of fragmentation was investigated. Extensively fragmented chondral tissue with a pastelike appearance showed superior extracellular matrix production and histological scoring compared with cartilage tissue minced into larger fragments (8, 2, or 1 mm).^4^ A hand-mincing device was tested that did not show a positive biological effect on chondral tissue but simplified and accelerated the mincing process.^11^ Harvesting and mincing human cartilage tissue with an arthroscopic shaver has only recently been investigated in vitro: Evuarherhe et al^8^ found no difference in the viability, proliferation capacity, and extracellular matrix production of cells isolated from minimally manipulated and shaver-minced cartilage samples from 6 fresh-frozen cadaveric donors.

In contrast, a higher viability of cells derived from minced cartilage tissue compared with a control group of cells derived from minimally manipulated cartilage was observed in the present study. The longer time of cell culture (42 days) in the study of Evuarherhe et al^8^ is a possible reason for the different findings. Concerning the reported proteoglycan proportion, Evuarherhe et al found no difference between and within the investigated subgroups at different time points. The observations of the present study are similar to the findings of Evuarherhe et al in comparing proteoglycan proportions between chondrocytes isolated from minced and minimally manipulated cartilage tissue.

Investigations on the effect of PRP on chondrocytes have led to previous contradictory findings. The application of lyophilized PRP on isolated chondrocytes in an in vitro model showed a dose- and time-dependent positive effect on cell proliferation and viability.^10^ However, the comparability with these in vitro findings on isolated chondrocytes is limited because MCI deals with cartilage tissue consisting of chondrocytes and an extracellular matrix instead of isolated chondrocytes, and the PRP used was not autologous but a lyophilizate of 12 donors. The effect of PRP on MCI was investigated in an animal study. Repeated injections of PRP as an adjuvant to MCI for treating full-thickness cartilage lesions of the knee were compared with MCI alone. The investigation did not show a difference in macroscopic scoring or in the proportion of hyaline and fibrocartilage in the lesions.^14^ Our investigation did not indicate any effects of PRP on the cell outgrowth, proliferation capacity, or viability of cells derived from shaver-minced cartilage tissue. However, chondrocytes derived from the MCP group showed an increased ability to produce proteoglycans after a chondrogenic stimulus compared with chondrocytes derived by CR, which does not account for chondrocytes derived from shaver-minced cartilage tissue without PRP augmentation. The observed statistically significant difference in the proteoglycan proportion of chondrocyte spheroids in the MCP group compared with chondrocyte spheroids in the CR group was evident in 8 of 10 investigated sample pairs. However, in absolute numbers, the difference in the proteoglycan proportion was subtle. It remains uncertain whether this in vitro observation demonstrates that PRP supplementation leads to enhanced cartilage regeneration in vivo.

MCI and ACI are the only cell-based cartilage repair techniques currently available. The advantage of MCI compared with ACI is its lower cost, as there is no need for biomimetic scaffolds and cell processing under regulatory guidelines.^3^ Additionally, MCI can be performed as a 1-step procedure. However, clinical evidence on the efficacy of MCI is limited to a few studies and short-term outcomes.^9^ ACI, on the other hand, has been thoroughly investigated. Several randomized controlled trials comparing ACI with bone marrow stimulation procedures have documented the beneficial long-term effects of ACI.^6,15,19^ Furthermore, the ACI procedure controls for patient-specific cell quality because chondrocyte isolation and in vitro cell expansion, proliferation capacity, and viability of chondrocytes are tested and reported before reimplantation. In contrast, controlling the quality and regenerative potential of individual transplanted cartilage tissue during MCI is limited because of the 1-step character of the procedure. In this context, the high correlation of cell outgrowth, proliferation capacity, and cell viability between cartilage tissue taken by CR and shaver-minced or PRP-augmented cartilage tissue from the same donors is important to be considered. These observations indicate that during MCI, donor-specific factors have a higher effect on the cell outgrowth, proliferation capacity, and cell viability of transferred cartilage tissue than shaver mincing or PRP augmentation. This means that significant individual differences in the quality of transplanted cartilage tissue must be assumed during MCI.

### Limitations

The present in vitro study shows encouraging results regarding the usability of an arthroscopic shaver and PRP during MCI. However, the significance concerning the in vivo efficacy of the procedure is limited; in particular, the transferability of the presented in vitro results to an in vivo situation as well as the primary stability of the minced cartilage graft, resistance to mechanical stress, and integration into surrounding cartilage tissue need to be further investigated. A study linking our findings on chondrocyte proliferation and functionality during MCI to clinical outcomes would be of utmost interest, especially considering the significant donor-specific differences that were observed in this in vitro investigation. The present study was not designed to investigate certain donor-specific factors that influence chondrocyte functionality during MCI. Ultimately, the present study cannot replace a controlled clinical trial comparing MCI with an established cartilage repair technique to obtain profound statements on the efficacy of MCI.

## Conclusion

This study showed that harvesting and mincing human cartilage tissue using an arthroscopic shaver were feasible when considering chondrocyte survival and functionality. This was supported by the observation of a higher viability of cells derived from shaver-minced cartilage compared with those derived from CR. Augmentation with PRP did not influence cell outgrowth or the viability of cells isolated from transferred cartilage tissue but increased the normalized proteoglycan content of examined chondrocyte spheroids. This indicates that augmentation with PRP during MCI increased the ability of transferred chondrocytes to produce a cartilage-specific extracellular matrix.
